# Heat Shock Protein HSP27 Secretion by Ovarian Cancer Cells Is Linked to Intracellular Expression Levels, Occurs Independently of the Endoplasmic Reticulum Pathway and HSP27's Phosphorylation Status, and Is Mediated by Exosome Liberation

**DOI:** 10.1155/2017/1575374

**Published:** 2017-02-23

**Authors:** Matthias B. Stope, Gerd Klinkmann, Karoline Diesing, Dominique Koensgen, Martin Burchardt, Alexander Mustea

**Affiliations:** ^1^Department of Urology, University Medicine Greifswald, Ferdinand-Sauerbruch-Strasse, 17475 Greifswald, Germany; ^2^Department of Gynaecology and Obstetrics, University Medicine Greifswald, Ferdinand-Sauerbruch-Strasse, 17475 Greifswald, Germany

## Abstract

The heat shock protein HSP27 has been correlated in ovarian cancer (OC) patients with aggressiveness and chemoresistance and, therefore, represents a promising potential biomarker for OC diagnosis, prognosis, and treatment response. Notably, secretion of soluble HSP27 has been described by a few cell types and may take place as well in OC cells. Therefore, we studied HSP27 secretion mechanisms under diverse cellular conditions in an OC cell model system. Secretion of HSP27 was characterized after overexpression of HSP27 by transfected plasmids and after heat shock. Intra- and extracellular HSP27 amounts were assessed by Western blotting and ELISA. Protein secretion was blocked by brefeldin A and the impact of the HSP27 phosphorylation status was analyzed overexpressing HSP27 phosphomutants. The present study demonstrated that HSP27 secretion by OVCAR-3 and SK-OV-3 cells depends on intracellular HSP27 concentrations. Moreover, HSP27 secretion is independent of the endoplasmic reticulum secretory pathway and HSP27 phosphorylation. Notably, analysis of OC cell-born exosomes not only confirmed the concentration-dependent correlation of HSP27 expression and secretion but also demonstrated a concentration-dependent incorporation of HSP27 protein into exosomes. Thus, secreted HSP27 may become more important as an extracellular factor which controls the tumor microenvironment and might be a noninvasive biomarker.

## 1. Background

Ovarian cancer (OC) therapy is restricted to a great extent by a limited understanding of the driving force of OC breakout and progression. OC represents the eighth most common female malignancy and first most common cause of death amongst gynaecological malignancies in the Western hemisphere [1]. However, an unsatisfactory characterization of the underlying molecular and cellular dynamics primarily prevents the development of new diagnosis and treatment approaches.

Heat shock proteins (HSP) are cellular factors whose expression levels are frequently upregulated in carcinoma tissue with respect to the nonmalignant tissue counterpart. Recently, accumulating evidence has emphasized the pivotal role of HSP in initiation and progression of cancer. Cytoprotective and thus frequently prooncogenic activities of HSP are particularly in demand when cellular proteins are disordered. Moreover, HSP are mostly stress-inducible cell survival factors that are upregulated by chemical and physical stresses (e.g., oxidative stress, hypoxia, and heat shock) as well as radio- and chemotherapy. HSP functions are closely linked to bind specifically to client proteins and subsequently to control turnover and activity of these proteins [2, 3]. While the two most abundant HSP group members, HSP70 and HSP90, have been investigated most extensively, only little is known about the small HSP family member, HSP27, and its effect in OC progression.

Elevated levels of HSP27 are associated with treatment resistance and poor prognosis in numerous types of malignancies including breast, prostate, liver, and gastric carcinoma [4–9]. HSP27 function in OC progression, however, has not been studied yet in detail. In 1995, Langdon and coworkers have determined HSP27 levels in primary OC tissue samples and correlated expression levels of the protein with the aggressiveness and chemoresistance of OC [10]. Recently, histological studies have shown that elevated HSP27 levels are important predictors of peritoneal metastasis [11, 12]. Furthermore, an experimental approach utilizing an established OC cell line revealed that HSP27 activity governs resistance to paclitaxel treatment [13]. It is known that HSP27's prooncogenic capacity is due to its properties as a cell survival factor; consequently, HSP27 represents a promising biomarker for OC diagnosis and treatment benefit.

Notably, a few studies have demonstrated that HSP27 is liberated by a so far unknown cellular machinery [14, 15] and that secreted HSP27 also may exhibit paracrine properties in tissues microenvironment. Secreted HSP27 was found to induce angiogenesis and to take effect in the regulation of NFКB signaling [16–18]. Moreover, secreted HSP27 suppresses the release of proinflammatory factors while activating the production of anti-inflammatory factors [19]. In blood samples from OC patients, increased levels of HSP27-specific antibodies were described suggesting the presence of the corresponding antigen and secreted HSP27 protein itself [20]. Zhao and coworkers have shown an increase of circulating HSP27 in serum samples from OC patients exclusively in peritoneal metastasis-positive patients, meanwhile the authors failed to demonstrate differences between the groups of healthy women and metastasis-negative OC patients [12].

The objective of the present study was to examine HSP27 secretion by OC cells against the background of future applications in gynecooncological diagnosis and prognosis. Thus, analysis of intra- and extracellular HSP27 levels was performed in an OC cell culture model.

## 2. Methods

### 2.1. Cell Culture

The human OC cell lines OVCAR-3 and SK-OV-3 were both received from Cell Lines Service (Eppelheim, Germany) and propagated in RPMI 1640 medium (Biochrom, Berlin, Germany) containing 10% foetal bovine serum (Biochrom), 0.125% gentamicin (Ratiopharm, Ulm, Germany), and 0.1% insulin (Novo Nordisk, Mainz, Germany) and DMEM/F12 (Life Technologies, Darmstadt, Germany) supplemented with 5% foetal bovine serum and 0.125% gentamicin, respectively. Cells were passaged twice a week and maintained in a humidified atmosphere at 37°C and 5% CO_2_.

### 2.2. Proliferation Assay

The proliferation of OC cells was evaluated utilizing a CASY Cell Counter and Analyzer Model TT (Roche Applied Science, Mannheim, Germany). Briefly, adherent cells were detached with trypsin at different time points, suspended at 1 : 100 dilution into CASYton buffer (Roche Applied Science) and 400 *µ*l of cell suspension was analyzed in triplicate. Measurement was performed using a capillary of 150 *µ*m in diameter and cell line specific gate settings were used to discriminate between living cells, dead cells, and cellular debris (OVCAR-3: 9.00 *µ*m/15.75 *µ*m; SK-OV-3: 7.00 *µ*m/15.15 *µ*m).

### 2.3. Enzyme Linked Immunosorbent Assay (ELISA)

HSP27 concentration in cell culture supernatant was determined by use of the DuoSet ELISA kit human HSP27 (DY1580; R&D, Minneapolis, MN, USA) with Substrate Reagent Pack containing stabilized hydrogen peroxide (Part #895000; R&D) and stabilized tetramethylbenzidine (Part #895001; R&D) according to the manufacturer's instructions. Duplicates of each sample were measured. Cells and cellular debris were sedimented (1, 3 ×g, 5 min), the supernatant was incubated with capture antibodies (overnight, 4°C), and soluble HSP27 was determined using a BMG FLUOstar OPTIMA Microplate Reader with OPTIMA software 2.10 (BMG Labtech, Offenbach, Germany).

### 2.4. Western Blotting Analysis

Intracellular proteins were extracted from the OC cell lines by lysing the samples in Laemmli buffer (50.0 mM Tris-HCl, pH 6.8, 2.0% SDS, 10.0% (w/v) glycerol, 0.01% bromophenol blue, and 5.0%  *β*-mercaptoethanol). Equal amounts of cell lysate were separated by SDS-PAGE using 10% polyacrylamide (Biorad, Munich, Germany) and blotted onto a polyvinylidene fluoride membrane. Subsequently, the membrane was blocked (1 h, 5.0% nonfat dry milk in 50 mM Tris, 150 mM NaCl, and 0.01% Tween 20) and incubated with primary antibodies directed against HSP27 (1 : 10,000), *β*-actin (1 : 40,000), and glyceraldehyde-3-phosphate dehydrogenase (GAPDH; 1 : 10,000; all primary antibodies from Cell Signaling Technology, Danvers, USA) overnight at 4°C. After washing, blot membranes were incubated with dye-conjugated mouse- and rabbit-specific secondary antibodies (1 : 10.000; LI-COR Biotechnology, Lincoln, NE, USA) for 1 h. Protein signals were visualized by enhanced chemiluminescence with a LI-COR Odyssey Infrared Imager System (LI-COR Biotechnology) according to the manufacturer's instructions. Protein levels of HSP27, *β*-actin, and GAPDH were quantified using the Image Studio Lite Version 4.0 software (LI-COR Biotechnology). *β*-Actin signals served as loading control for lysates; GAPDH was the loading control in exosome preparations. HSP27 expression levels were normalized to controls (control = 1.0).

### 2.5. Overexpression of HSP27 and HSP27 Phosphomutants

24 h before transfection OC cells were harvested by trypsinization and replated at a density of 3.0 × 10^5^ cells/well into a 6-well cell culture plate containing the appropriate medium without foetal bovine serum and gentamicin. 3.0 *μ*g of plasmid DNA/well (pHSP27 wt, pHSP27-3A, pHSP27-3D [21]; empty control vector pcDNA3.1, Life Technologies) was transfected into OVCAR-3 cells using Lipofectamine 2000 (Life Technologies) according to the manufacturer's instructions. Transiently transfected cells were harvested 24 h, 48 h, and 72 h after transfection and analyzed by Western blotting and ELISA.

### 2.6. Heat Shock Experiments

For heat shock experiments SK-OV-3 cells were plated at a density of 1.5 × 10^5^ cells/well into a 6-well cell culture plate and after an incubation of 24 h two protocols of heat shock were performed. Cells were exposed to a temperature program (heat shock cycle: 37°C for 24 h, 39°C for 24 h, and 37°C of 24 h) or cells were uniquely exposed to 39°C for 4 h. Control cells were treated at 37°C for 72 h and 4 h, respectively.

### 2.7. Inhibition of the Endoplasmic Reticulum Secretory Pathway

Cells were plated at a density of 1.5 × 10^5^ cells/well into a 6-well cell culture plate containing the appropriate medium. After 24 h brefeldin A was supplemented at a final concentration of 80 nM using ethanol vehicle as control. At indicated time points, cells as well as cell culture supernatant were harvested and intracellular and extracellular HSP27 concentrations were determined by Western blotting and by ELISA analysis.

### 2.8. Exosome Preparation

Preparation of exosomes was followed as previously described [22]. Briefly, cells were incubated in T75 cell culture flasks with 20 ml cell culture medium and exposed to the heat shock cycle. Subsequently, cell culture supernatant was harvested and cleared by centrifugation (100 ×g; 5 min; 4°C), and exosomes were pelleted by ultracentrifugation (100,000 ×g; 1 h; 4°C; Sorvall RC M120 GX Micro-Ultracentrifuge with Ultra-Clear Centrifuge Tubes Beckman, 11 × 34 mm). Exosome sediments were resuspended in Laemmli buffer and analyzed by Western blotting.

### 2.9. Statistics

The statistical analysis was performed using SPSS 13.0 software (SPSS, Chicago, IL, USA). Data were evaluated using the graphics and statistics software program Graph Pad Prism 5.01 (GraphPad Software, La Jolla, CA, USA). Statistical comparisons were performed using Student's *t*-test with *P* ≤ 0.05 (*∗*), *P* ≤ 0.01 (*∗∗*), and *P* ≤ 0.001 (*∗∗∗*) given as significant.

## 3. Results

### 3.1. HSP27 Expression in OVCAR-3 and SK-OV-3 Cell Lines Is Correlated with Differentially Secreted HSP27 Protein Levels

**Figure 1:**
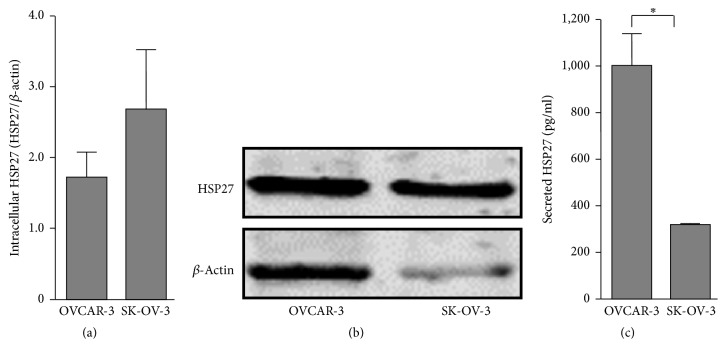
Basal intracellular expression and secretion of HSP27 in OVCAR-3 and SK-OV-3 cells. (a) OVCAR-3 and SK-OV-3 cells were propagated and relative intracellular HSP27 expression was determined by Western blotting. HSP27 signal was normalized to *β*-actin. (b) Secreted HSP27 concentrations were determined in OVCAR-3 and SK-OV-3 cell culture supernatants by ELISA. Graphs of samples were calculated as the mean ± SD. *P* values were determined by Student's *t*-test with *P* ≤ 0.05 (*∗*) given as significant.

Secretion of the low molecular weight protein HSP27 from OC tissue has been described previously [12, 20]. However, further studies are required to unravel the cellular circumstances that promote HSP27 secretion with all of the attendant effects on tumor microenvironment control. In context of this, we aimed at analyzing basal expression of HSP27 in OVCAR-3 and SK-OV-3 cells by Western blotting (Figures 1(a) and 1(b)). Intracellular HSP27 amount in SK-OV-3 cells (HSP27/*β*-actin: 2.71 ± 0.80) was only slightly enhanced compared to OVCAR-3 cells (HSP27/*β*-actin: 1.82 ± 0.28, *P* = 0.1425). Notably, the subtle distinction between intracellular HSP27 expressions in both cell lines was linked with significantly elevated levels of secreted HSP27 protein in OVCAR-3 cells (Figure 1(c)). In OVCAR-3 cell culture supernatant, a significant increase in secreted HSP27 protein (1,004.9 ± 137.4 pg/ml) compared to SK-OV-3 cells (322.5 ± 0.9 pg/ml; *P* = 0.0197) has been exhibited.

### 3.2. Upregulation of Intracellular HSP27 Expression Leads to Enhanced Secretion of the Protein

**Figure 2:**
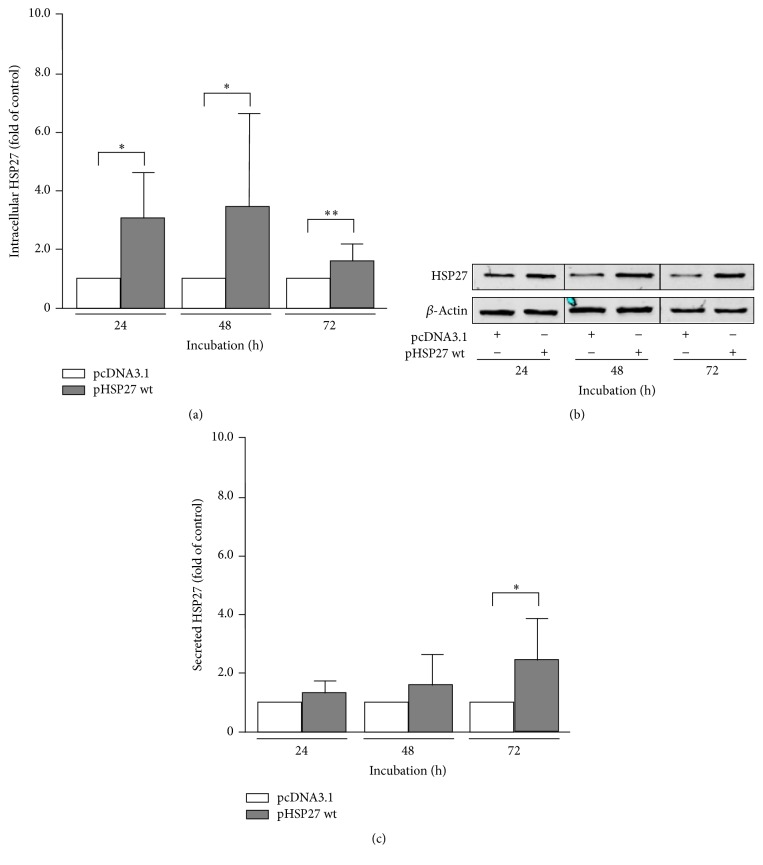
Intracellular expression and secretion of HSP27 in OVCAR-3 cells transfected with the overexpression plasmid pHSP27 wt. (a) OVCAR-3 cells were transfected with 3.0 *µ*g pHSP27 wt plasmid and empty control vector pcDNA3.1, respectively. Relative intracellular HSP27 expression was determined by Western blotting at indicated time points. HSP27 signal was normalized to *β*-actin. (b) Representative Western blots of experiments as described in (a). (c) Secreted HSP27 concentration in supernatants of OVCAR-3 cells determined by ELISA at indicated time points. Cells were treated as described in (a). Graphs of samples were calculated as the mean ± SD. *P* values were determined by Student's *t*-test with *P* ≤ 0.05 (*∗*) and *P* ≤ 0.01 (*∗∗*) given as significant.

Genetically engineered overexpression and heat shock induction of intracellular HSP27 protein have been used in order to explore a potential connection of intracellular HSP27 expression and secreted HSP27 levels. As shown in Figures 2(a) and 2(b), transfection of OVCAR-3 cells with the DNA plasmid pHSP27 wt led to a clear overexpression of HSP27 over a period of 72 h (24 h: 3.12 ± 1.38, *P* = 0.0131; 48 h: 3.50 ± 2.90, *P* = 0.0203; 72 h: 1.68 ± 0.48, *P* = 0.0041) compared to pcDNA3.1 transfected controls. Parallel determination of HSP27 protein in the cell culture supernatant by ELISA demonstrated elevated amounts of secreted HSP27 compared to control transfections (24 h: 1.25 ± 0.58, *P* = 0.3625; 48 h: 1.68 ± 0.95, *P* = 0.1506; 72 h: 2.38 ± 1.28, *P* = 0.0418) (Figure 2(c)).

**Figure 3:**
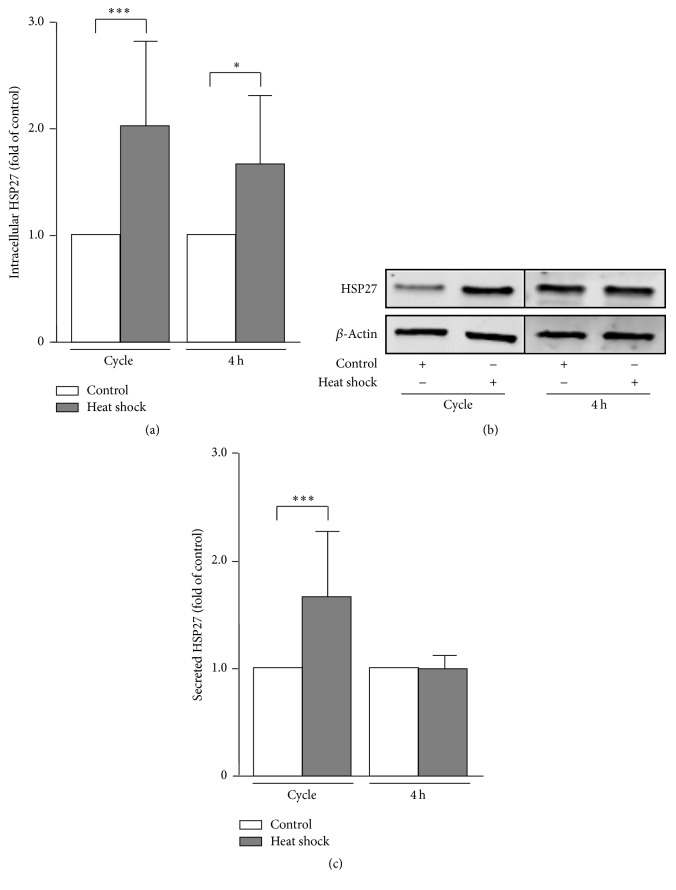
Intracellular expression and secretion of HSP27 in heat shock-treated SK-OV-3 cells. (a) SK-OV-3 cells were treated with a 37°C/39°C/37°C heat shock cycle for 24 h each temperature (cycle) and a unique 39°C heat shock for 4 h (4 h), respectively. Cells incubated by 37°C for 72 h and 4 h, respectively, served as controls. Relative intracellular HSP27 expression was determined by Western blotting. HSP27 signal was normalized to *β*-actin. (b) Representative Western blots of experiments as described in (a). (c) Secreted HSP27 concentration in supernatants of SK-OV-3 cells determined by ELISA. Cells were treated as described in (a). Graphs of samples were calculated as the mean ± SD. *P* values were determined by Student's *t*-test with *P* ≤ 0.05 (*∗*) and *P* ≤ 0.001 (*∗∗∗*) given as significant.

To verify these data, a comparable approach was used in which the endogenous HSP27 expression in SK-OV-3 cells was activated by heat shock induction. A 37°C/39°C/37°C heat shock cycle for 24 h each temperature (cycle) and a unique 39°C heat shock for 4 h (4 h) were performed and analyzed by Western blotting (Figures 3(a) and 3(b)). In both experimental setups, HSP27 levels rose significantly (cycle: 2.10 ± 0.72, *P* = 0.0037; 4 h: 1.71 ± 0.61, *P* = 0.0315) compared to 37°C control incubations. As shown in Figure 3(c), elevated intracellular HSP27 levels during heat shock cycle were linked to the significant increase in HSP27 secretion (cycle: 1.80 ± 0.42, *P* = 0.0001). After 4 h heat shock, however, no changes in secreted HSP27 concentrations were detectable (4 h: 0.99 ± 0.12, *P* = 0.8258).

### 3.3. HSP27 Secretion Is Insensitive to Brefeldin A Treatment

**Figure 4:**
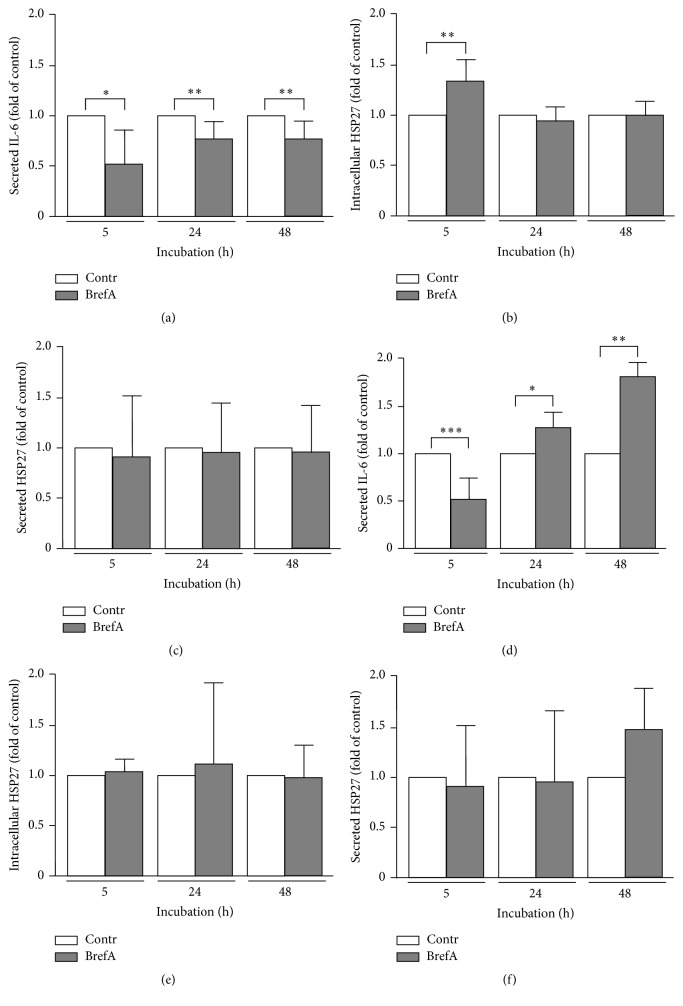
Intracellular expression of HSP27 and secretion of IL-6 and HSP27 in OVCAR-3 and SK-OV-3 cells in the presence of brefeldin A. (a, b, and c) OVCAR-3 cells were incubated with 80 nM brefeldin A and IL-6 secretion (a), intracellular HSP27 (b), and secreted HSP27 (c) were determined by ELISA (a and c) and Western blotting (b), respectively, at indicated time points. (d, e, and f) SK-OV-3 cells were incubated with 80 nM brefeldin A and IL-6 secretion (d), intracellular HSP27 (e), and secreted HSP27 (f) were determined by ELISA (d and f) and Western blotting (e), respectively, at indicated time points. Intracellular HSP27 signal was normalized to *β*-actin. All values were standardized to vehicle incubated controls (control = 1.0). Graphs of samples were calculated as the mean ± SD. *P* values were determined by Student's *t*-test with *P* ≤ 0.05 (*∗*), *P* ≤ 0.01 (*∗∗*), and *P* ≤ 0.001 (*∗∗∗*) given as significant.

The molecular route through endoplasmic reticulum and Golgi apparatus to the extracellular space is the main secretory pathway for secreted proteins in eukaryotic cells and sensitive to inhibition by brefeldin A [23, 24]. To proof whether endoplasmic reticulum secretory pathway functionality may be critical for basal HSP27 secretion, we performed brefeldin A incubation experiments using interleukin 6 (IL-6) as positive control (Figures 4(a) and 4(d)). As expected, IL-6 secretion was suppressed in OVCAR-3 cells compared to vehicle treated cells over a period of 48 h (5 h: 0.53 ± 0.29, *P* = 0.0497; 24 h: 0.73 ± 0.16, *P* = 0.0056; 48 h: 0.76 ± 0.18, *P* = 0.0086) (Figure 4(a)). In SK-OV-3 cells, however, IL-6 secretion was blocked after 5 h (0.48 ± 0.19, *P* < 0.0001) followed by significantly increased levels of secreted IL-6 after 24 h and 48 h of incubation (24 h: 1.28 ± 0.19, *P* = 0.0278; 48 h: 1.65 ± 0.32, *P* = 0.0065) (Figure 4(d)).

Except the 5 h incubation of OVCAR-3 cells, brefeldin A treatment did not affect intracellular levels of HSP27 neither in OVCAR-3 cells (5 h: 1.30 ± 0.20, *P* = 0.0048; 24 h: 0.97 ± 0.21, *P* = 0.7389; 48 h: 1.00 ± 0.19, *P* = 0.9567) (Figure 4(b)) nor in SK-OV-3 cells (5 h: 1.02 ± 0.08, *P* = 0.6342; 24 h: 1.09 ± 0.77, *P* = 0.7599; 48 h: 0.98 ± 0.26, *P* = 0.8327) (Figure 4(e)). ELISA analysis of cell supernatant of OC cells propagated in the presence of brefeldin A revealed no alterations of secreted HSP27 in OVCAR-3 (5 h: 0.92 ± 0.54, *P* = 0.7686; 24 h: 0.97 ± 0.39, *P* = 0.9141; 48 h: 0.98 ± 0.34, *P* = 0.9013) (Figure 4(c)) and SK-OV-3 cells (5 h: 0.95 ± 0.57, *P* = 0.8097; 24 h: 0.98 ± 0.68, *P* = 0.9534; 48 h: 1.45 ± 0.47, *P* = 0.1040) (Figure 4(f)) compared to controls.

### 3.4. HSP27's Phosphorylation Status Does Not Control Protein's Secretion

**Figure 5:**
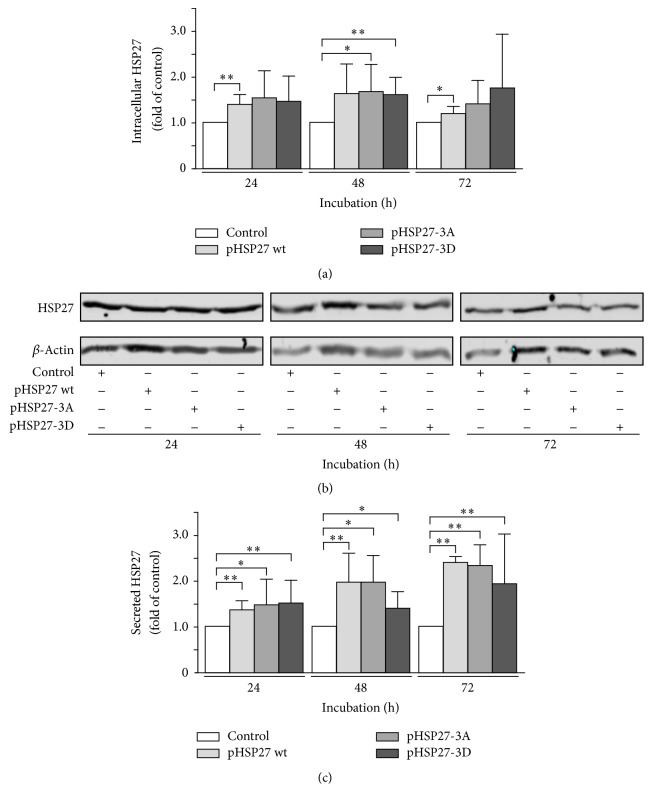
Intracellular expression and secretion of HSP27 in OVCAR-3 cells transfected with the overexpression plasmids pHSP27 wt, pHSP27-3A, and pHSP27-3D. (a) OVCAR-3 cells were transfected with 3.0 *µ*g pHSP27 wt, pHSP27-3A, pHSP27-3D plasmid, and empty control vector pcDNA3.1, respectively. Relative intracellular HSP27 expression was determined by Western blotting at indicated time points. HSP27 signal was normalized to *β*-actin. (b) Representative Western blots of experiments as described in (a). (c) Secreted HSP27 concentration in supernatants of OVCAR-3 cells determined by ELISA at indicated time points. Cells were treated as described in (a). Graphs of samples were calculated as the mean ± SD. *P* values were determined by Student's *t*-test with *P* ≤ 0.05 (*∗*) and *P* ≤ 0.01 (*∗∗*) given as significant.

Protein phosphorylation governs the oligomerization state of HSP27 and thus may be an initial step in following HSP27 transport into the extracellular space. Phosphorylated species of HSP27 exist in the form of monomers and low molecular weight oligomers and, therefore, are predominantly available for cellular protein secretion [25]. Transfection experiments using plasmids encoding for wild-type HSP27, the nonphosphorylable triple substitution mutant HSP27-3A (substitution of serine-15, serine-78, and serine-82 with alanine-15, alanine-78, and alanine-82), and the phosphomimicking triple mutant HSP27-3D (substitution of serine-15, serine-78, and serine-82 with aspartic acid-15, aspartic acid-78, and aspartic acid-82) demonstrated a distinct overexpression of all of the three proteins in OVCAR-3 cells over a period of 72 h (Figures 5(a) and 5(b)). Subsequent analysis of secreted HSP27 proteins indicated no significant differences in protein secretion comparing HSP27 wt (24 h: 1.38 ± 0.25, *P* = 0.0093; 48 h: 1.99 ± 0.46, *P* = 0.0014; 72 h: 2.41 ± 0.86, *P* = 0.0064), the phosphomutants HSP27-3A (24 h: 1.50 ± 0.38, *P* = 0.0177; 48 h: 1.99 ± 0.68, *P* = 0.0115; 72 h: 2.35 ± 0.90, *P* = 0.0096), and HSP27-3D (24 h: 1.52 ± 0.30, *P* = 0.0043; 48 h: 1.41 ± 0.31, *P* = 0.0182; 72 h: 1.93 ± 0.60, *P* = 0.0085) (Figure 5(c)). Again, genetically engineered overexpression of HSP27 led to an increase of extracellular protein as shown before (Figure 2); however, differences in secretion linked to nonphosphorylable HSP27 protein (HSP27-3A), physiologically phosphorylated HSP27 protein (HSP27 wt), and phosphoimitating HSP27 protein by charge and steric characteristics of the aspartic acid residues (HSP27-3D) have not been detected.

### 3.5. HSP27 Secretion Is Performed by Exosome Particles

**Figure 6:**
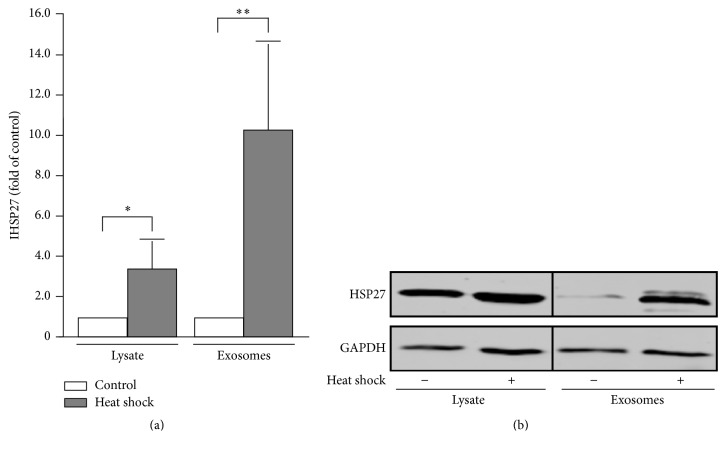
Load of HSP27 in exosomes from heat shock-treated SK-OV-3 cells. (a) SK-OV-3 cells were treated with a 37°C/39°C/37°C heat shock cycle for 24 h each temperature (heat shock) and compared to cells incubated by 37°C for 72 h (control). Relative intracellular HSP27 expression in cell lysates and relative HSP27 load in prepared exosomes was determined by Western blotting. HSP27 signal was normalized to GAPDH. (b) Representative Western blots of experiments as described in Figure 3(a).* P* values were determined by Student's *t*-test with *P* ≤ 0.05 (*∗*) and *P* ≤ 0.01 (*∗∗*) given as significant.

An alternative pathway for protein liberation is represented by shedding of membranous microvesicles. Within this group, there is one type of vesicles, exosomes, which are typically characterized as carrier vesicles primarily for proteins [26]. In case of SK-OV-3 cells a 37°C/39°C/37°C heat shock cycle induced the HSP27 expression intracellularly accompanied by enhanced HSP27 levels in extracellular exosomes (Figure 6). HSP27 protein analysis demonstrated a 3.43 ± 1.41-fold (*P* = 0.0135) induction of HSP27 expression in cell lysates and a 10.25 ± 4.47-fold (*P* = 0.0061) increase in HSP27 incorporation into extracellular exosomes (Figure 6(a)) as shown by Western blot detection of HSP27 normalized to GAPDH loading control (Figure 6(b)). Thus, these data not only confirmed the concentration-dependent correlation of HSP27 expression and secretion but also demonstrated a concentration-dependent incorporation of HSP27 protein into exosomal particles.

## 4. Discussion

Secretion of HSP27 has been detected in a few cell types including granulocytes, macrophages, platelets, and cells of the cardiovascular system [15, 19, 27, 28], as well as in malignant cells of lung, liver, breast, and ovary [12, 14, 29, 30]. Beyond that, the understanding towards cellular functionality of secreted HSP27 is fairly limited so far. In comparison to the well-characterized cell survival properties of intracellular HSP27 in mammalian cells, secreted HSP27 may harbor new functions, for example, in angiogenesis, inflammation, and vital signal transduction pathways [16–19, 28].

In the present study we could demonstrate that HSP27 secretion depends, amongst other factors, on intracellular HSP27 concentrations. The experimental induction of HSP27 expression by an overexpression plasmid as well as by heat shock led to elevated intracellular levels of the protein followed by an enhanced liberation of cell-free HSP27. Analysis of basal HSP27 expression and secretion in both cell lines, however, referred to further and, more likely, to individual mechanisms driving HSP27's secretion. Even though intracellular protein levels of HSP27 are very similar in OVCAR-3 and SK-OV-3 cells (Figure 1(a)), HSP27 secretion by OVCAR-3 cells was significantly higher compared to SK-OV-3 cells (Figure 1(c)). Moreover, significantly elevated and comparable overexpression of HSP27 over a period of 72 h as shown in Figure 2(a) demonstrated a time-dependent accumulation of cell-free HSP27 in the cell culture supernatant. It took 72 h to increase extracellular HSP27 protein significantly during overexpression experiments (Figure 2(c)). Taken together, HSP27 secretion by OC cells seems to be controlled by several mechanisms including the intracellular protein concentration. Furthermore, heat shock experiments with incubations for 24 h (heat shock cycle) and for 4 h (unique heat shock) at 39°C resulted in discrepancies in regard to the HSP27 secretion. While intracellular levels of the heat shock protein rose by both protocols, only cell culture supernatants of heat shock cycle treated cells exhibited increased concentrations of HSP27. A short-termed heat shock of 4 h may be insufficient to induce an intensified secretion of HSP27. In addition, it cannot be excluded that an incubation time of 4 h is too short for HSP27's significant accumulation in the cell culture supernatant.

The secretion of HSP27 has been already described in the literature; however, the pathway for HSP27 secretion is still unidentified. The N-terminus of HSP27 consists of the WDPF domain (tryptophan-aspartic acid-proline-phenylalanine) for oligomerization and a partially conserved region with so far unknown function. HSP27's C-terminus is defined by the *α*-crystallin oligomerization domain, and a flexible region which has been suggested to interact with client proteins [31]. A typical endoplasmic reticulum localization signal peptide of up to 50 hydrophobic amino acids is missed. Therefore, HSP27 transport via the endoplasmic reticulum appears unlikely and thus, endoplasmic reticulum inhibition by brefeldin A as shown in this study failed to block the secretion of HSP27.

The block of IL-6 liberation in the presence of brefeldin A served as positive control and demonstrated an unexpected increase in IL-6 secretion after 24 h restricted to SK-OV-3 cells (Figure 4(d)). As brefeldin A is a potent inhibitor of proinflammatory IL-6 secretion it is frequently utilized for flow cytometric determination of intracellular IL-6 [32]. Despite the unknown underlying mechanism, it is, to the best of our knowledge, the first report of this effect and may reflect OC cells heterogeneity in cellular response to chemical compounds.

Under physiological conditions, HSP27 is predominantly localized to the cytoplasm. Nevertheless, Wong et al. have shown that the phosphorylation of HSP27 may cause HSP27's translocation into the nucleus [33], suggesting an inducible HSP27-specific transport machinery. Moreover, the phosphomimetic HSP27 mutant HSP27-3D overexpressed in osteoblasts caused HSP27 accumulation in the endoplasmic reticulum, whereas wild-type HSP27 and the nonphosphorylable HSP27 mutant HSP27-3A were retarded in the cytosol [34]. Molecular functionality of HSP27 phosphorylation may explain the potential impact of this modification to protein secretion. HSP27 serves as a substrate for various kinases, for example, protein kinase B (PKB, Akt) [35], protein kinase C (PKC) [36], protein kinase D1 (PKD1) [37], and mitogen-activated protein kinase- (MAPK-) activated protein kinase 2 (MK2) [38]. In OC cells, HSP27 is primarily phosphorylated at serine S-15, S-78, and S-82 by the p38 MAPK-MK2 axis [39]. On the molecular level, HSP27 phosphorylation determines the oligomerization state of HSP27. Unphosphorylated HSP27 can form large oligomers up to 800 kDa, which are mainly linked with cell survival and cytoprotection. On the other hand, protein phosphorylation causes disassembly of HSP27 complexes and leads to formation of HSP27 mono-, di-, and tetramers revealing potentially distinct properties in cell fate control [25]. Tokuda et al. have demonstrated that collagen induces intracellular HSP27 phosphorylation in platelets and that phosphorylated HSP27 molecules are primarily secreted into the extracellular space [28]. Secreted HSP27 in plasma, however, is predominantly unphosphorylated [15], which may subsequently occur after release by cell surface and/or soluble phosphatases [40]. Thus, control of HSP27 oligomerization and thereby determination of HSP27 complexes' architecture and size possibly play pivotal role in HSP27 secretion regulation. However, overexpression experiments carried out with nonphosphorylable HSP27-3A and phosphomimicking HSP27-3D mutants in this study negated regulation of HSP27 secretion by phosphorylation in case of OC cells.

While IL-6 secretion was significantly blocked in the presence of the endoplasmic reticulum secretory pathway inhibitor brefeldin A, HSP27 expression and secretion were not affected. Surprisingly, for periods of brefeldin A incubation longer than 5 h, IL-6 levels in SK-OV-3 cell culture supernatants increased. This effect may reflect differences in OVCAR-3 and SK-OV-3 cell response to the cytotoxic agent brefeldin A. HSP27 secretion, however, could not be inhibited by brefeldin A, pointing to a differing secretion pathway for HSP27 release.

Protein secretion may also occur by direct translocation through the cytoplasmic membrane or by uptake into different types of export vesicles including exosomes [41]. In prostate cancer cells, HSP27 protein is unspecifically incorporated into exosomes [22], and in OC cells, Dolo et al. identified a protein release by cellular vesicle shedding [42]. The data presented here explicitly demonstrated the incorporation of HSP27 into exosomes, thereby liberating the protein from OC cells by bypassing the classical endoplasmic reticulum secretory pathway. From literature data, extracellular HSP27 appears to play a role in angiogenesis and inflammation, as well as in general signaling processes during tumor progression [16, 17, 19, 43]. Apart from that, HSP27's properties in the tumor microenvironment are widely unclear and thus representing the focus of recent research.

## 5. Conclusion

The tumor progression factor HSP27 is liberated from OC cells by exosomes. Protein's secretion appears independently of the phosphostatus as well as independently of the classical endoplasmic secretion pathway. Notably, HSP27 liberation is correlated with the intracellular HSP27 levels. This should be taken into consideration when anticancer treatment may induce HSP27 [3] and subsequently HSP27-mediated extracellular effects.

Regardless of the so far unknown functionality of extracellular HSP27 in OC cells and adjacent tumor tissues, secreted HSP27 may become a promising biomarker for OC. Zhao and coworkers compared 24 blood samples from healthy women to blood samples from 48 patients with epithelial OC. An association of elevated blood levels of secreted HSP27 with metastatic OC was found [12], but because of a low case number the study was of limited power. Nevertheless, secreted HSP27 may become more important as a cancer progression-related factor that controls tumor microenvironment activities. Thus, secreted HSP27 could be potentially useful as noninvasive biomarker for diagnosis, prognosis, and treatment response not only in OC therapy.
